# The Emergence of Up and Down States in Cortical Networks 

**DOI:** 10.1371/journal.pcbi.0020023

**Published:** 2006-03-24

**Authors:** David Holcman, Misha Tsodyks

**Affiliations:** 1 Department of Mathematics, Weizmann Institute of Science, Rehovot, Israel; 2 Department of Neurobiology, Weizmann Institute of Science, Rehovot, Israel; 3 Departement d'etudes cognitives, Ecole Normale Superieure, Paris, France; University College London, United Kingdom

## Abstract

The cerebral cortex is continuously active in the absence of external stimuli. An example of this spontaneous activity is the voltage transition between an Up and a Down state, observed simultaneously at individual neurons. Since this phenomenon could be of critical importance for working memory and attention, its explanation could reveal some fundamental properties of cortical organization. To identify a possible scenario for the dynamics of Up–Down states, we analyze a reduced stochastic dynamical system that models an interconnected network of excitatory neurons with activity-dependent synaptic depression. The model reveals that when the total synaptic connection strength exceeds a certain threshold, the phase space of the dynamical system contains two attractors, interpreted as Up and Down states. In that case, synaptic noise causes transitions between the states. Moreover, an external stimulation producing a depolarization increases the time spent in the Up state, as observed experimentally. We therefore propose that the existence of Up–Down states is a fundamental and inherent property of a noisy neural ensemble with sufficiently strong synaptic connections.

## Synopsis

The cerebral cortex is continuously active in the absence of sensory stimuli. An example of this spontaneous activity is the phenomenon of voltage transitions between two distinct levels, called Up and Down states, observed simultaneously when recoding from many neurons. This phenomenon could be of a critical importance for working memory and attention. Thus, uncovering its biological mechanism could reveal fundamental properties of the cortical organization. In this theoretical contribution, Holcman and Tsodyks propose a mathematical model of cortical dynamics that exhibits spontaneous transitions between Up and Down states. The model describes an activity of a network of interconnected neurons. A crucial component of the model is synaptic depression of interneuronal connections, which is a well-known effect that characterizes many types of synaptic connections in the cortex. Despite its simplicity, the model reproduces many properties of Up–Down transitions that were experimentally observed, and makes several intriguing predictions for future experiments. In particular, the model predicts that the time that a network spends in the Up state is highly variable, changing from a fraction of a second to more than ten seconds, which could have some interesting implications for the temporal characteristics of working memory.

## Introduction

In the absence of sensory inputs, cortical neural networks can exhibit complex patterns of intrinsic activity. The origin of this spontaneous activity in the cortex is still unclear, but recent studies have reported some important characteristics of the dynamics. For example, it has been demonstrated [3] that the spontaneous population activity in the visual cortex is highly correlated with organized patterns, called orientation map, occurring usually during some specific visual stimulation. In a parallel direction, the studies [1] using two-photon calcium imaging and [2] extracellular recordings have reported that single neuron membrane potentials in slice preparations can spontaneously transit between two states, called the Up and Down states. The origin of this phenomenon, originally described in [4] and [5], is not precisely known, but the transitions have been ascribed to the intrinsic network property [6]. Transitions are almost abolished by pharmacological blockers such as glutamate receptor antagonists [1,2] and totally abolished by glutamate and GABA receptor antagonists [1]. As a consequence, the Up and Down state transitions are a good example of how a connected network exhibits a complex correlated dynamic in the absence of any external inputs.

In previous models based on [7], the Up to Down state transition was attributed to the effect of neuronal adaptation due the building up activity of *K* ^+^ channels. In [8], numerical simulations are presented, where the somatic voltage of a neural population shows an Up and Down state activity. However, no arguments are given to explain why there is a unique Up state and why this Up state is stable. Actually, the authors “hypothesized that recurrent synaptic excitation produces a network bistability with an active Up state and an inactive Down state,” where the *I_K−NA_* current is responsible for the switching between states. Their picture of the Up and Down state dynamics is explained by a hysteresis loop, which requires a current injection for a specific range of the parameters.

We propose here to investigate the role of the spontaneous activity in the generation of the Up and Down state transitions, using a modeling approach. We attribute the spontaneous fluctuations to the external noise at the level of neural populations. We demonstrate that in a sufficiently interconnected network which shows activity-dependent synaptic depression, two nonsymmetric attractors emerge. Due to the particular geometry of those attractors, the noise generates a bistability regime (two-state regime) but this is achieved in a very different way, compared with the case of symmetric attractors. When inhibitory connections are included into the model, the results are not changed qualitatively, thus to extract the main ingredients of the Up–Down states phenomena, we have decided to restrict the present analysis to a model containing only excitatory connections. Due to the activity-dependent synaptic depression, which was observed in neocortical slices [9], the network dynamic does not reach a saturation value. Our model is formulated with a reduced set of two equations (Equation 1) describing the mean activity of a homogeneous population of neurons. The first variable represents the mean activity of the whole population and can be scaled to represent locally the average membrane potential, while the second variable is the mean rate of synaptic depression in the network. This simplified “mean-field” description allows us to analyze the network in the low-dimensional space of these two variables (phase space).

Our analysis reveals that for certain values of the parameters of the model that characterize the strength of network connectivity, the phase space of the network contains two stable fixed points (attractors) that correspond to the stable stationary values of activity and depression of the network. One of the attractors corresponds to the state of zero activity, and the other one corresponds to higher activity. The basin of attraction associated with the first fixed point (all the initial values of the variables that dynamically flow to this fixed point) corresponds to the Down state of activity, and the basin of attraction of the second fixed point corresponds to the Up state of activity. Noise activity produces random transition between the basins, which generates the Up–Down state dynamics. The model predicts in particular that transition to the Up state is always associated with a spike generated in the network population.

## Results

### Dynamics of the Network

The analysis of the deterministic autonomous dynamical system 1 described in Materials and Methods (when σ = 0 and *I(t)* = 0) reveals that for small values of *w_T_*, the phase diagram of the system contains one stable fixed point only, corresponding to zero value of activity (P1: *V* = 0, *μ* = 1). At some minimal value of *w_T_*, two more fixed points appear: one saddle point (P3, see Figure 1) and another one—repulsor. If the synaptic strength *w_T_* becomes even larger, at a certain critical value *w_c_* of the average synaptic weight, a Hopf bifurcation occurs, the repulsor becomes an attractor (P2, see Figure 1), and an unstable limit cycle appears around it (Figure 1A). This analysis was carried out in [13,15] and is briefly recapitulated in Protocol S1. Intuitively, the Hopf bifurcation occurs when recurrent connections are sufficiently strong to overcome synaptic depression to produce enough recurrent excitation to sustain a persistent Up state. Numerically, *w_c_* can be estimated as the value *w_T_* where the real part of the linearized matrix of the system 1 crosses 0 from negative to positive, at the critical point *P*_2_. Since the dynamical system is smooth near the Up state attractor, the existence of the limit cycle *C*, i.e., the periodic solution to the network equations (Equation 1) corresponding to oscillatory behavior of the network, is guaranteed by the standard theory of Hopf's bifurcation (see [16]). The critical point *P*_1_ = (0,1) is always an attractor of the system. To study the role of noise in a depressing neural network, the equations in Equation 1 are analyzed using the phase diagram of the system, represented in Figure 1. Figure 1B represents a trajectory in the phase space. Due to the noise activity, the mean potential V can escape from the basin of attraction of the point *P*_1_. This phenomena is the well-known exit problem for a dynamical system perturbed by random noise in general [17,18] and for nonuniformly random noise perturbations in a few cases [19]. When the deterministic system has two basins of attraction, the noise (which only appears in one variable) can lead to transitions between the two states. When the network dynamics relax in the Down state, it approaches closer to the separatrix, where a small fluctuation due to the noise might be enough to produce a transition. However, this transition across the separatrix is not sufficient to produce an Up state, because this region is characterized by an unstable limit cycle. Consequently, further noise fluctuation is necessary to push the network inside the Up state region (which is defined as the region inside the unstable limit cycle, see Figure 1A). In this intermediate region, between the separatrix and the unstable limit cycle, the dynamic is mainly driven by the deterministic part, leading to a fast rotation around the unstable limit cycle. This rotation generates a fast spike of the population voltage. In the more realistic model, the population spike would correspond to a synchronous discharge of many neurons. This peculiar property of the network dynamics was analyzed in detail in [14], where it was shown that the underlying reason for the emergence of population spikes is the separation of time scales between the voltage dynamics and synaptic depression (*t_r_≫τ*). Under this condition, the population spikes have amplitudes that are much larger than the voltage in the Up state, and a duration that is much faster than the typical duration of Up and Down states.

**Figure 1:**
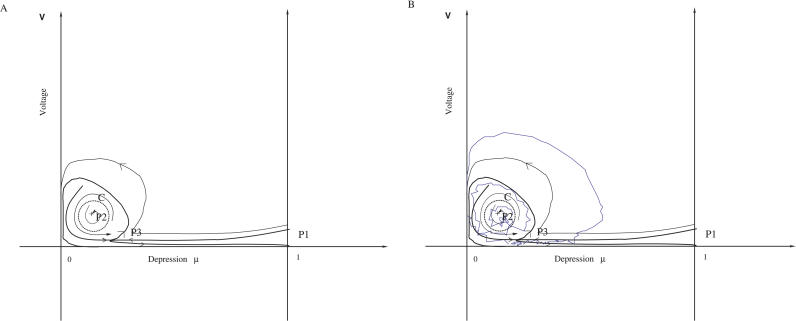
Schematic Representation of the Phase Space (*μ,V*) (A) The depression *μ* is plotted on the *x*-axis, while the variable *V* is on the *y*-axis (for the schematic representation, no scale is given). The recurrent sets are the attractor points *P*_1_ (the Down state), the saddle point *P*_3_ **,** and the attractor *P*_2_ (the Up state), separated by an unstable limit cycle C (dashed line). The region inside the unstable limit cycle is the basin of attraction of *P*_2_. By definition, it is the Up state. For a specific value of the parameter, an homoclinic curve can appear. The unstable branch of the separatrix starting from *P*_3_ terminates inside the basin of attraction of the Down state. The noise drives the dynamics beyond the separatrices (lines with arrow converging to *P*_3_) but a second transition is necessary for a stochastic trajectory to enter inside the Up state delimited by C. However a single noise transition is enough to destabilize the dynamics from the Up to the Down state. (B) Example of a trajectory in the phase space.

This spike generation in the network population always occurs during a transition from a Down to an Up state, which is a prediction of our model. When the rotation is performed in the phase space, any noise fluctuation can push the dynamics inside the Up state region. The two consecutive transitions are reasonably probable because compared with the amplitude of the noise, the separatrices are located very close to the unstable limit cycle C, as can be seen from a more careful analysis of simulations (unpublished data).

Figure 2A shows the results of simulations in the case when the deterministic system has two basins of attraction (*w_T_ > w_c_*). The parameters of the model are presented in Table 1 (for these values of parameters, numerical analysis outlined above produces *w_c_* ≈ 10.3). The voltage difference between the Up and Down state is about 15 mV. Such value is approximated by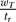 , as can be seen by finding the steady-state solutions of Equation 1. The population activity in the Down state is much lower than in the Up state but still non-zero, as was observed experimentally (see, e.g., [27]).

**Figure 2:**
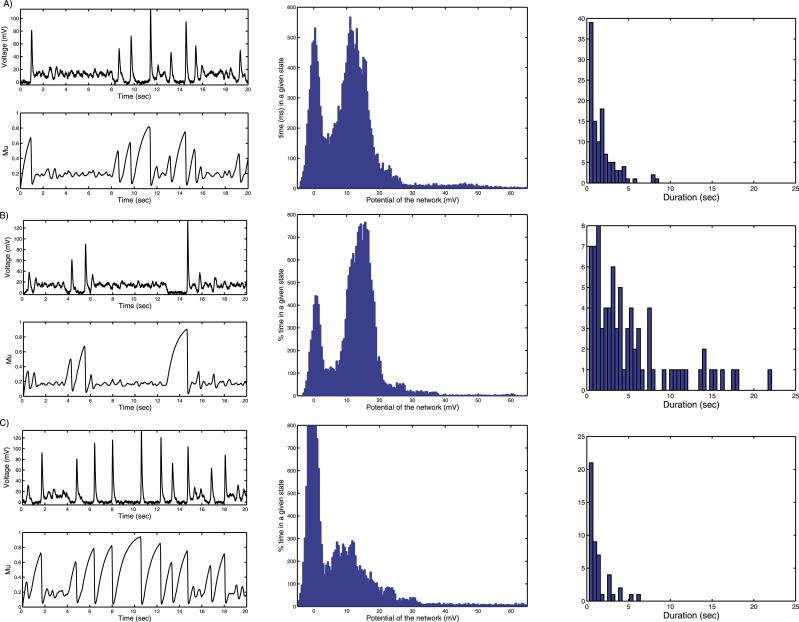
Up–Down State Dynamics under Normal and Clamp Conditions (A) A typical realization of Up–Down transition dynamics in the stochastic model with *w_T_* = 12.6. Top graph: (Up) average population voltage (mV), baseline at 0 mV; (Down) plot of the depression variable *μ* as a function of time: when the system arrives in the Down state, it is depressed. Then the depression recovers slowly toward 1. Usually the noise is able to produce a transition, before full recovery is achieved. Bottom graph: Histogram of the population voltage: fraction of the time spent in a given state. Units are in ms on the *y*-axis and in mV on the *x*-axis: the fraction of total time spent in the Up and Down state is about the same during the 20 s of simulations. The maximum time in the Up state is about 500 ms but the mean maximum time is about 3 s. Right column graph of (A): histogram of the duration of the Up states. (B) Effect of an external stimulation: depolarization. When a constant external input (I = 0.8) is added into the model, the neural network spends on average more time in the Up state than in Down state. In that case, the attractor point *P*_1_ is shifted and is now closer to the separatrix. (C) Hyperpolarization, simulated by a current injection*.* A hyperpolarization (I = −0.3) drives the ensemble of neurons at a lower mean potential, and as a result the neural network spends on average more time in the Down state.

**Table 1:**
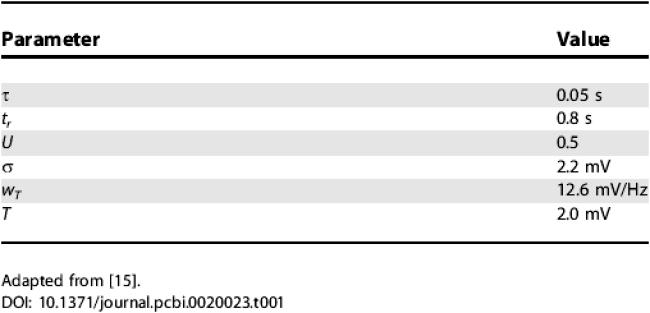
Values of Parameters Used in the Simulation

The time course of the voltage is comparable to the Up–Down state dynamics observed experimentally [1,2], and in particular each transition from Down to Up is associated with a spiking event. This peak of synchrony was reported using two-photon calcium imaging and it was associated with the time when nearby neurons fire [1], revealing a transition from a Down to an Up state.

The transition from the Down to the Up state can be understood as follows: the noise can lead the system to cross the border between the basin of attractions (the separatrix), but not necessarily the limit cycle. In that case, a fast spiking event is generated, as can be observed in Figure 2A, when the dynamic comes back into the Down state. When the dynamic succeeds in getting into the Up state, by a second transition due to noise, it stays there for a certain mean time, which can be approximated by the mean first passage time to the unstable limit cycle. Once the limit cycle is crossed, the dynamic relaxes exponentially to the Down state. This analysis indicates that the consecutive Up state durations are independent from each other, as conjectured in [20]. Note also that the network undergoes oscillations during the Up state due to the presence of the limit cycle around the attractor P2 (see above). Similar oscillations in the Up state have been observed in the potential trace of single neuron recordings from the barrel cortex of anesthetized rats (I. Lampl, personal communication). Similarly, under whole cell recording of layer 2/3 pyramidal neuron of the barrel cortex, large fluctuations of the membrane potential in the Up state were also reported in [21]. Since there is no limit cycle around the attractor P1, no oscillations are observed while the network is in the Down state.

### Effects of External Inputs on Up–Down Dynamics

We study the sensitivity of the Up–Down dynamics to changes in the average value of the external input. To this end, we repeated the simulations of the network dynamics for different average values of *I* (see Equation 1). The results of these simulations are shown in Figure 2A in the absence of any external input. Adding a positive input, which is the equivalent of adding a current injection that depolarizes the cells, increases the time spent in the Up state compared with the control case, as seen in Figure 2B. This result is compatible with the experimental studies, where a similar shift was reported in the response to sensory inputs [22–24]. In contrast, adding negative input changes the dynamics in the opposite direction, to the point where the Up state disappears completely, as shown in Figure 2C. We also computed the distribution of the durations of the individual Up state epochs for these three cases (Figure 2, column 3). Surprisingly, we observed the presence of exceptionally long Up state epochs, as reflected in the long tail of the distribution. To complete the analysis, we show the dependency of the mean duration of the Up state and the frequency of Down–Up transitions as a function of external input (Figure 3). While the mean duration increases with the input, as expected, the transition frequency has a unique maximum.

**Figure 3:**
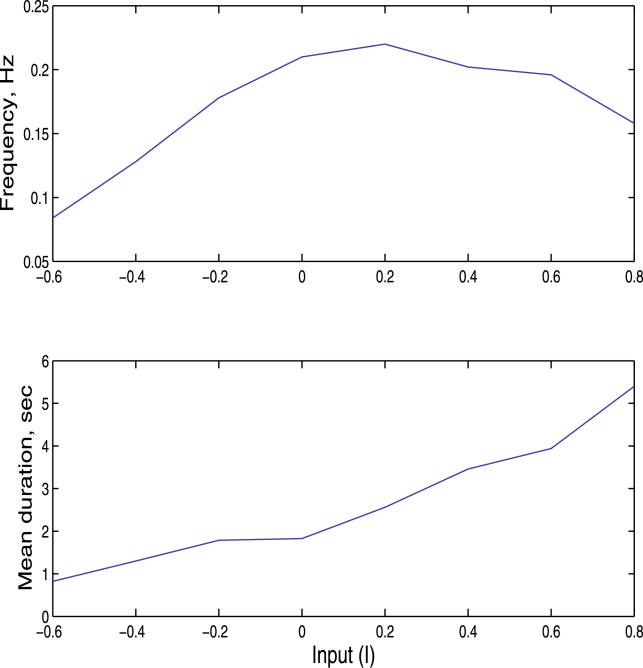
Frequency and Duration of the Mean Time Spent in the Up State as a Function of I The top graph represents the frequency of Up states as a function of Input I. Hyperpolarizing the neurons is generated for *I* < 0, while depolarization is generated for *I* > 0. The result suggests that there is a unique value of I for which the frequency of the Up state is maximal. In the bottom graph, the mean duration of the Up states is plotted as a function of I.

### Effects of Changing the Total Synaptic Connections

When the total synaptic weight *w_T_* is changed, it affects the Up and Down state dynamics: increasing *w_T_* increases the mean time spent in the Up state and its activity level (Figure 4A), while decreasing *w_T_* reduces the time spent in the Up state and the activity level (Figure 4B). Increasing *w_T_* can be interpreted as the result of long-term potentiation (LTP), which is known to reinforce the synaptic connections. Qualitatively similar effects were obtained when we changed the value of *U* (unpublished data). Since changes in *w_T_* are usually associated with the postsynaptic mechanism of LTP, while changes in *U* are associated with the presynaptic mechanism [25,26], these results show that both mechanisms have similar effects on the distribution of network activity between the Up states and Down states.

**Figure 4:**
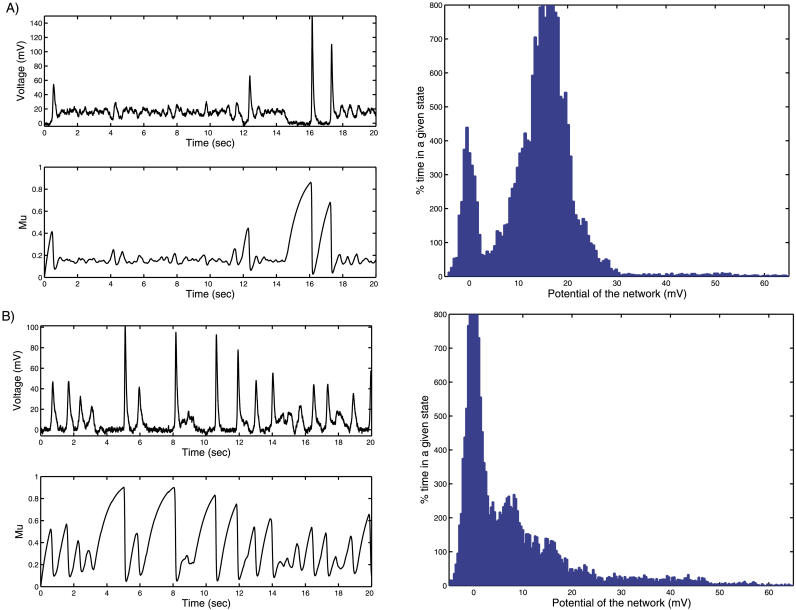
Effect of Synaptic Plasticity on Up–Down State Dynamics (A) Effect of increasing *w_T_*, which is equivalent to the LTP effect. The effect of LTP is obtained by increasing the total synaptic connections *w_T_* to a value of 15. The effect is similar to a depolarization injection current. (B) Effect of LTD on Up–Down state dynamics*.* The effect of LTD is obtained by decreasing the total synaptic connections *w_T_* to a value of 11. The effect is similar to hyperpolarizing the ensemble of neurons.

### Interaction between the Network State and the Stimulus

The functional significance of the spontaneous dynamics is revealed by considering the effect on the cortical response to sensory stimulation. Several experimental studies [21,24,27] reported that neurons respond more strongly if the stimulus arrives during the Down state of activity. To check whether our model can reproduce this effect, we applied brief excitatory pulses with the frequency of 1 Hz and computed the average voltage response separately for stimuli that fell on Up and Down states of the network. The resulting responses are shown in Figure 5. As one can see, the response is much weaker for the Up state, because the synapses are very depressed, making the interactions within the network weaker. If the amplitude of the stimulus, applied during the Up state, is increased, it may trigger a transition to the Down state (unpublished data). This effect was also predicted in [28] and observed experimentally in [2].

**Figure 5:**
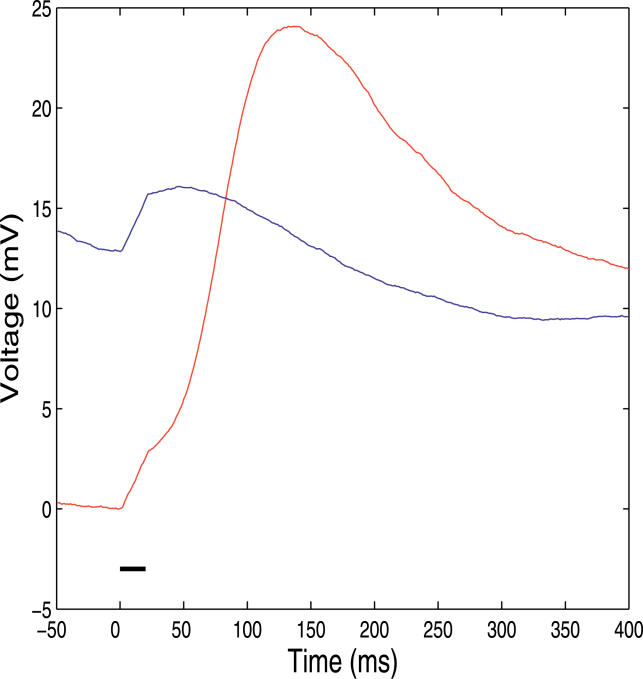
Average Response to a Brief Excitatory Pulse Which Produces a Small Depolarization in Each State The average voltage response is computed. The blue curve shows the response in the Down state. The red curve shows the response in the Up state. The horizontal black bar indicates the duration of the stimulus (starting at *t* = 0 ms). The time response is different in each state, and in agreement with the experimental results of [27].

## Discussion

We conclude that the Up–Down state dynamics can be generated from a stochastic dynamical system that describes the mean activity of a recurrent excitatory network with synaptic depression. Depending on the average *w_T_* strength of connections, the dynamical system presents various recurrent sets (Figure 1) that include two stable attractors. The two attractors appear only when a minimal synaptic strength *w_c_* is reached. In the absence of synaptic depression, the Up state could only exist at the level of saturation (i.e., at a very high firing rate). This follows easily from the equations, and can be understood intuitively, too, since without depression nothing would prevent the system with recurrent excitation to increase its activity until it reaches saturation. Thus, dynamic fluctuations in the synaptic weights that are due to activity-dependent synaptic depression counterbalance the activity fluctuations and prevent them from growing in amplitude. The role of the intrinsic noise is to produce stochastic transitions from one attractor to another. Thus, if the noise amplitude is small, spontaneous transitions will be rare, but the Up state could still be evoked by an external trigger. This phenomenology could be relevant for working memory. We do not know how to convert *w_c_* into a precise measurable quantity, such as the minimum number of synaptic connections that will guarantee the existence of the two states. The model that we presented here is the “mean-field” approximation for the average activity of a large network of spiking neurons. As shown in the literature, it usually provides a qualitatively good description of the network activity (see, e.g., [14]). In order to have a significant source of noise in the mean-field model (after averaging), the network should receive a spatially correlated noise, e.g., due to common input to different neurons.

### Transition from the Different States

As predicted in the model, the Up and Down state transition of the network activity from one basin of attraction to another is the result of the noise activity. Since even in the Down state, the network is far from equilibrium (the synapses are always depressed), the mean time the network spends in the Down state is not approximated by the mean first passage to the separatrix, starting near the attractor of the Down state, rather it is comparable to the mean time it takes for the synapses to recover from a certain depressed activity. This deterministic relaxation is not much affected by how strong the noise is. In the Down state, the synaptic depression fluctuates much more than in the Up state. As long as the network is in the Down state, the depression recovers exponentially, until a transition in the Up state is produced.

A transition from the Up to the Down state is achieved when the noise pushes the dynamics outside the Up state region. The mean time spent in the Up state is the mean first passage time from any point inside of the basin of attraction to the unstable limit cycle and is exponentially long as a function of the amplitude of the noise [18,29]. Thus, when the noise amplitude is changed in the numerical simulations, the time spent in the Up state changed (unpublished data).

When the noise amplitude is increased, the time spent in the Up state decreases and more transition between states occurs. As a consequence, the Up states occurring in slices (where the noise amplitude is supposed to be less than in vivo) should last longer than in vivo.

The present model does not include any channel activity, which affects the dynamics of the membrane potential. Activation of *K^+^* conductances in neostriatal spiny neurons was suggested to produce a stable Up state [4], and this conductance activity has been used in some models [8] to explain the transition from the Up to the Down state. Other factors, such as feedback inhibition and synaptic facilitation, could also be involved in the Up–Down state dynamics. However, in our case, we find it remarkable that many of the experimental observations about Up–Down dynamics can be reproduced here within the reduced model, suggesting that synaptic depression could be a dominating mechanism.

### Steady State of the Network and Synaptic Depression

Analysis of Figure 2A (lower left) shows that synapses are highly depressed most of the time. This suggests that even in the absence of external stimuli, the synaptic noise is sufficient to keep the system far from equilibrium, including the Down state. It is interesting to note that synapses are not functioning near equilibrium, but far from the steady state. As a consequence, the firing rate driven by the noise activity produces synaptic depression. Interestingly, the Up and Down states do not disappear if weak inhibition is added by simulating the extra inhibitory population with linear synapses from and to the excitatory one (unpublished data), rather we found that the main role of inhibition is to decrease the mean time that the system stays in the Up state.

The assumption that synaptic depression can be a possible mechanism underlying the Up and Down state dynamics has already been suggested in [21,24]. As a possible prediction of the model, using the results shown in Figure 4, when *w_T_* increases to a certain value, we expect that the Up and Down state dynamics should appear in an ensemble of neurons, as a result of LTP induction. In the opposite direction (Figure 4B), any decrease in the total synaptic weight should lead to an equilibrium state where the Up and Down states completely disappear. We predict that an intermediate stage should appear, where the ensemble generates synchronous spikes, but cannot sustain any Up states. It is conceivable that LTP experiments will increase local connections among only a few neurons, which will generate an Up and Down state activity only for them.

### Up State and Adaptation to Large Stimuli

Because in the Up state the response of the network to external stimulus is weaker compared with the Down state (see Figure 5), it is conceivable that a neuron makes a transition in the Up state to decrease the amplitude of the response to a large number of action potentials. This transition can be seen as a possible mechanism for adaptation: by decreasing the response in the Up state, the network filters some spikes, but it continues to generate action potential. If the network had stayed in the Down state, a similar spiking activity would have led to a large response, possibly to a saturated regime. Because in the Up state, the mean depression μ is lower than in the Down state, individual neurons become synaptically isolated from the rest of the network. This mechanism of isolation in an Up state provides a possible explanation for the smaller response of individual neurons to stimulus in the Up state compared with the Down state. This was shown theoretically in Figure 5 and described experimentally in [21]). This mechanism is also helpful in understanding the lack of response propagation in the network, as reported in [21].

This transition process can be compared with the phenomena of adaptation in cone photoreceptors, appearing when light is increased by several orders of magnitude: cone photoreceptors can continue to modulate light, where usually without adaptation the photoresponse would have been negligible. Conceivably, the dynamics associated with synaptic depression in the Up state is a mechanism of adaptation and has a global effect on the voltage sensitive channels, regulating the firing rate in the Up state.

### Processing Information with Up and Down States

In [1], it was reported that independent or weakly coupled small neuronal ensembles generate Up and Down state dynamics. Subsets of neurons are in an Up state while others are in a Down state. Refinement of the present model can be made by considering many neuron ensembles which present each Up and Down state dynamics, and by adding a correlation term in the system of Equation 1 to correlate each independent subsets of neurons. In that case, the temporal sequence of activation of each subnetwork can code information in a more robust manner, rather than would be the case for a single neuron. Thus, those subsets of neurons where Up and Down states are observed may be considered as a second integrated structure, beyond the level of individual neurons. As a consequence, subnetworks that present Up and Down states might be needed to guarantee reliable storage of information. The genesis of such subnetworks might also be considered at an intermediate level between synaptic and cortical plasticity.

## Materials and Methods

We consider a neural network connected by excitatory connections, described by the mean firing rate, which is the average of the firing rate over the population. We do not present here the equations where inhibition connections are added to the model. The change of the mean activity is modeled as a stochastic process. Neurons are connected with depressing synapses, and the state of a synapse is described by the depression parameter *μ* [10,12]. The mean activity of such a network can be approximated by the following dynamical system [11]


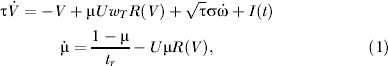


where *V* is an average synaptic input (measured in mV with a base line at 0 mV), *w_T_* measures the average synaptic strength in the network, *I(t)* is an external input, *U* and *t_γ_* are utilization parameter and recovery time constants of synaptic depression (see [12–14] for a derivation of the auditory cortex). The first term on the right-hand side of the first equation of Equation 1 represents the effect of the intrinsic decay. The second term on the right side represents the synaptic input, which is scaled by the synaptic depression parameter *μ*. The third term on the right side summarizes all uncorrelated sources of noise, with amplitude σ. *R(V*) is an average firing rate (in Hz) given by the threshold-linear dependence on the voltage:


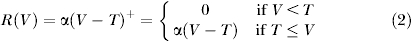


where *T* > 0 is a threshold and *α* = 1*HZ/mV* is a conversion factor. The second equation in Equation 1 describes the activity-dependent dynamics of the synaptic depression parameter according to the phenomenological model proposed in [12]. Briefly, every incoming spike leads to abrupt decrease in the instantaneous synaptic efficacy (by a utilization factor *U*) due to depletion of the neurotransmitter. Between the spikes, synaptic efficacy recovers to its original state (*μ* = 1) with the time constant *t_r_*.

## Supporting Information

Protocol S1. Fixed Points Analysis Click here for additional data file. (2 KB TEX)

## Abbreviations

LTP: long-term potentiation
